# Plant–Insect Interactions on Aquatic and Terrestrial Angiosperms from the Latest Albian (Early Cretaceous) of Estercuel (Northeastern Spain) and Their Paleoenvironmental Implications

**DOI:** 10.3390/plants12030508

**Published:** 2023-01-22

**Authors:** Artai A. Santos, Luis M. Sender, Torsten Wappler, José B. Diez

**Affiliations:** 1 Departamento de Xeociencias Mariñas e Ordenación do Territorio, Facultade de Ciencias do Mar, Universidade de Vigo, 36310 Vigo, Spain; 2 Centro de Investigación Mariña, Universidade de Vigo (CIM-UVIGO), 36310 Vigo, Spain; 3 Fundación Conjunto Paleontológico de Teruel-Dinópolis/Museo Aragonés de Paleontología, 44002 Teruel, Spain; 4 Department of Natural History, Hessisches Landesmuseum Darmstadt, Friedensplatz 1, 64283 Darmstadt, Germany; 5 Section Palaeontology, Institute of Geosciences, Rheinische Friedrich-Wilhelms-Universität Bonn, 53115 Bonn, Germany

**Keywords:** plant–insect interactions, angiosperms, paleoecology, insect behaviour, Early Cretaceous, Iberian Peninsula

## Abstract

Fossils of plant–insect interactions are direct evidence of paleoecological relationships between these two dominant groups in terrestrial ecosystems. We present a variety of plant–insect interactions from the late Early Cretaceous (latest Albian) in the Estercuel locality in northeastern Spain (Iberian Peninsula), affecting two types of terrestrial angiosperms and the basal eudicot *Klitzschophyllites*, which is one of the oldest putative members of aquatic Ranunculales found to date. The study of these interactions revealed 23 different damage types belonging to eight functional feeding groups (hole feeding, margin feeding, skeletonization, surface feeding, piercing and sucking, mining, oviposition and galling), suggesting these angiosperms were an important source of food and lodging for insects in the Iberian ecosystems during the late Early Cretaceous. Notably, the diversity of damage in the leaves of angiosperms suggests a diverse community of herbivorous insects and a variety of strategies of interactions with plants at the end of the Early Cretaceous in the southwestern Tethys realm.

## 1. Introduction

Plants and insects are two dominant groups in terrestrial ecosystems. Their entangled life histories for the past 410 million years make their interactions an important source of information for understanding ecological and evolutionary processes in the development of terrestrial environments. Evidence for these plant–insect interactions dates almost from the origin of terrestrial vascular plants in the mid Silurian to Early Devonian [1,2,3,4,5,6,7]. Nevertheless, these relationships intensified during the Early Cretaceous, paralleling the diversification of angiosperms [6,8]. This change in the composition of plant communities during the Early Cretaceous marks the origin of the “Fourth Phase of the Herbivore Expansion”, the last of the phases of the expansion of insect herbivory in deep time based on the relationships between plants and insects [6,8]. This phase, beginning during the mid-Early Cretaceous, is marked by the complex patterns of ecological and evolutionary relations that resulted in a shift of plant–insect interactions from gymnosperms to angiosperms, which took place during the “26-million-year-long Aptian-Albian Gap” [5,8,9].

Although the number of studies on plant–insect interactions in the fossil record has increased recently, evidence for these interactions in angiosperms, particularly aquatic taxa, of the Lower Cretaceous during the “Aptian–Albian Gap” is sparse and overwhelmingly restricted to externally feeding (exophytic) functional feeding groups (FFGs) [10,11,12]. The Aptian–Albian record of plant–insect interactions is documented by the work of Filho et al. [13], who reported several damage types (DTs) from the Crato Formation (Aptian-Albian) in Brazil, where they identified margin feeding, oviposition, piercing and sucking, and galling on ferns and margin feeding, skeletonization, and galling on aquatic and non-aquatic angiosperms. 

Evidence of plant–insect interactions from Cretaceous deposits in Spain is very scarce so far and restricted to the Albian. A previous study documented evidence of herbivory on plants from the Lower Cretaceous in the Iberian Peninsula, including hole, marginal, and surface feeding on leaves of Nymphaeaceae (water lily family) from the upper Albian deposits at the top of the Utrillas Formation [12]. Some of the damages were interpreted as having been caused by leaf beetles (Coleoptera: Chrysomelidae). Recently, another publication on this topic focused on the interactions on leaves of Caytoniales (genus *Sagenopteris*) from the lower-middle Albian of the underlying Escucha Formation also in northeastern Spain [14]. In addition, plant–insect interactions on angiosperm leaves in this area have only been mentioned in previous works where the taxonomy of the floristic elements was studied [15,16,17,18]. 

In this work, we show that a variety of interactions were present between insect and angiosperm leaves, the latter including basal eudicotyledons from the latest Albian in northeastern Spain, including multiple DTs and FFGs, which indicate the diversity and intensity of these interactions on primitive angiosperms during the “Aptian–Albian Gap”, a formative interval in the ecological and evolutionary expansion of angiosperms. Consequently, the objectives of the present work are: (a) to describe the plant–insect interactions on the leaves of both terrestrial and aquatic angiosperms from a late Early Cretaceous (latest Albian) fossil site in the Iberian Peninsula; (b) to identify possible groups of arthropods that may be responsible for these interactions; and (c) to infer their corresponding environmental and ecological implications.

## 2. Geological and Stratigraphical Setting

**Figure 1:**
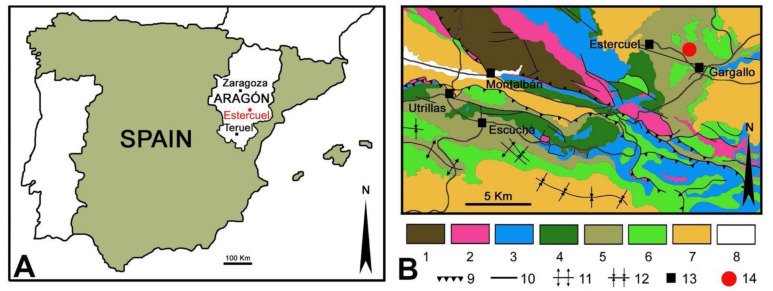
(**A**) Geographic map with location of the Estercuel locality in the Aragón region (Spain). (**B**) Geological map of the studied area and nearby setting. Legend of the map: 1 Carboniferous, 2 Triassic, 3 Jurassic, 4 Early Cretaceous, 5 Albian, 6 Late Cretaceous, 7 Oligocene–Miocene, 8 Quaternary, 9 Thrust belt, 10 Fault, 11 Anticline, 12 Syncline, 13 Localities, 14 Fossil site with insect–plant interactions. Both maps were modified from [21].

The leaves we have studied came from the “La Dehesa” fossil site near the village of Estercuel (Teruel, NE Spain, Figure 1A). The site is stratigraphically located at the upper part of the Utrillas Formation [19,20,21] within the Boundary Marls Unit (Figure 1B), composed of greyish to green laminated marls and claystones containing fossil plants with intercalated yellow and white sandstones and grey sandy claystones. The stratigraphic section containing the fossils comprises grey claystones intercalated with fine- to coarse-grained yellow sandstones [16,21]. The finest-grained lithologies yield macro- and microfloral assemblages composed of a variety of terrestrial and aquatic angiosperms as major clades, in addition to several taxa of conifers and scarce remains of ferns, quillworts (Isoetales), and some freshwater algae and a few dinoflagellates. These assemblages were deposited in a tidally influenced fluvial sedimentary environment that developed alongside freshwater coastal ponds and marshes [16].

**Figure 2:**
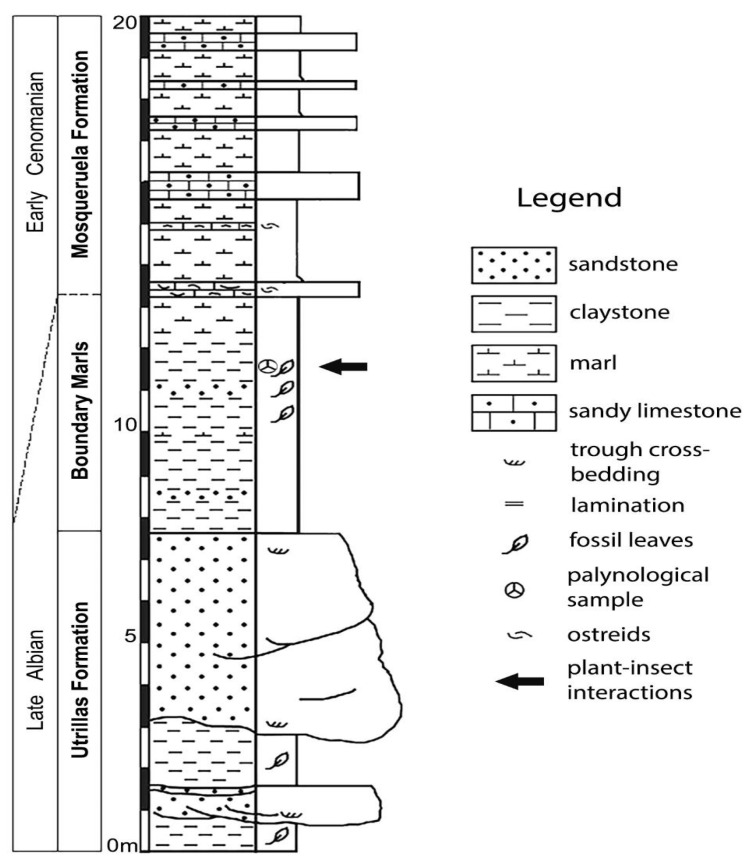
Stratigraphic section of the latest Albian “La Dehesa” fossil site in Estercuel locality (Spain) bearing the studied interactions (modified from [21]).

The Boundary Marls Unit at the fossil (Figure 2) site has been dated as latest Albian based on the stratigraphic distribution of palynomorph taxa from a palynological assemblage found in the same horizon where the studied plant–insect interactions occurred [16]. These taxa are *Afropollis jardinus* (Brenner) Doyle, Jardiné and Doerenkamp; *Stellatopollis barghoornii* Doyle; *Liliacidites doylei* Ward; *Liliacidites inaequalis* Singh; *Senectotetradites varireticulatus* (Dettmann) Singh; *Cyclonephelium chabaca* Below; *Microreticulatisporites sacalii* (Deák and Combaz) Ravn; *Gabonisporis pseudoreticulatus* Boltenhagen; *Elaterosporites klaszii* (Jardiné and Magloire) Jardiné; *Equisetosporites ambiguus* (Hedlund) Singh; *Impardecispora trioreticulosa* (Cookson and Dettmann); *Lophotriletes babsae* (Brenner); and *Tricolporoidites* sp.

## 3. Results

Twenty-three DTs were identified, belonging to eight FFGs (hole feeding, margin feeding, skeletonization, surface feeding, piercing and sucking mining, oviposition, mining and galling). The following DTs were identified on specimens of *Klitzschophyllites*: DT01, DT02, DT03, DT04, DT07, DT12, DT13, DT15, DT26, DT81, DT16, DT100, DT66, DT52 (14 DTs); on Angiosperm Type 1: DT01, DT02, DT03, DT04, DT05, DT13, DT14, DT15, DT16, DT17, DT29, DT35, DT43, DT46, DT62, DT120 (16 DTs); and on Angiosperm Type 2: DT01, DT02 and DT15 (3 DTs).

### 3.1. Interactions on Leaves of Genus Klitzschophyllites

#### 3.1.1. Hole Feeding

**Figure 3:**
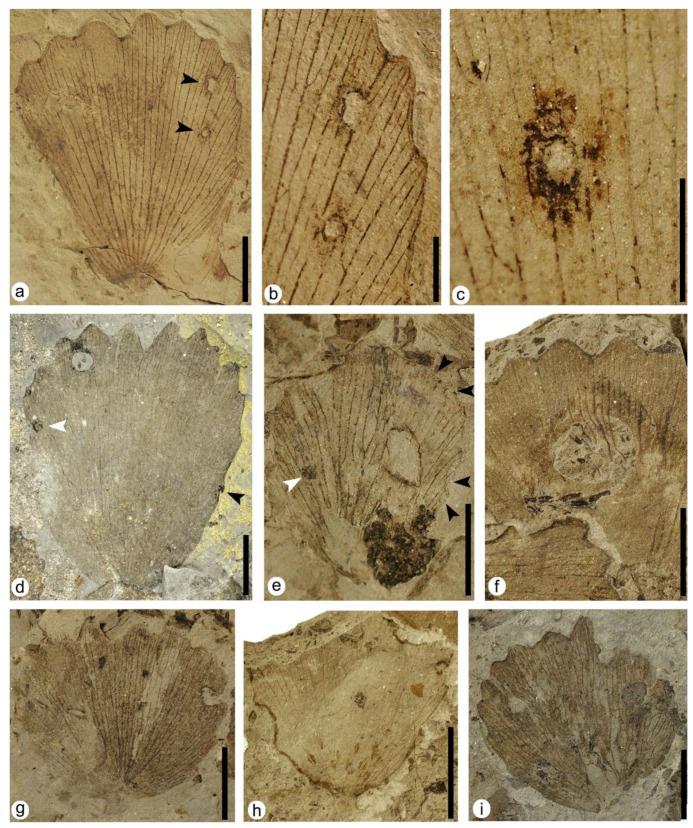
Plant–insect interactions on leaves of the aquatic angiosperm *Klitzschophyllites*. (**a**) MAP-8346, a complete leaf with hole feeding DT01 and DT02 indicated with black arrowheads. (**b**) Detail of the two holes DT01 and DT02 in photo (**a**). (**c**) Detail of the counterpart of the lower hole DT01 in photo (**b**) showing the thick reaction rim. (**d**) MAP-8347, a complete big leaf with hole feeding DT02, margin feeding DT12 (black arrowhead), and mining DT66 (white arrowhead). (**e**) MAP-8349, a nearly complete leaf with hole feeding DT03, margin feeding DT81 (black arrowheads) and galling DT52 (white arrowhead). (**f**) MAP-8353, with a big hole feeding DT04 showing strings of veins. (**g**) MAP-8355, with hole feeding DT07 and skeletonization DT16. (**h**) MAP-8354, with margin feeding DT12 and oviposition DT100. (**i**) MAP-8348, with hole feeding DT02 and margin feeding DT13 and DT26. Scale bars: (**a**,**d**,**f**–**i**) = 1 cm; (**b**,**c**) = 2 mm; (**e**) = 5 mm.

DT01: specimen MAP-8346 (Figure 3a–c). Leaf with two hole feedings, both located on the right margin of the leaf and of different sizes and shapes. The upper hole feeding in the upper right area is ellipsoidal to oval in shape, 0.9 mm in maximum length, containing a dark reaction rim variable in thickness from 0.06 mm to 0.19 mm wide. The interaction was not restricted by the veins, as it crosses at least three of them. The lower hole feeding in the upper right area is circular to subcircular in shape, 0.75 to 0.9 mm wide, presenting a black reaction rim with a thickness ranging from 0.06 mm to 0.15 mm. Although this hole crosses at least one of the leaf’s main veins, it seems limited by the thickest veins.

DT02: specimens MAP-8346 (Figure 3a,b), MAP-8347 (Figure 3d), MAP-8348 (Figure 3i). Hole feeding round in shape from 1.7 to 1.87 mm in maximum dimension, presenting a dark reaction rim from 0.06 mm to 0.22 mm in width. In specimen MAP-8348, this interaction crosses at least five veins and shows a dark patch of necrotic tissue extending from the upper part of the hole to the upper margin of the leaf, with dimensions of 1.12 mm long and up to 1.64 mm at its widest point. 

DT03: specimens MAP-8349 (Figure 3e), MAP-8350, MAP-8351, MAP-8352. Specimen MAP-8349 shows a prominent hole feeding in the central-right part of the leaf, with a rhomboid shape and a maximum size of about 3.12 mm long and 2.03 mm wide. Hole feeding crosses several veins of the leaf, although at the upper left margin in MAP-8349, apparently a thicker vein limited the expansion of the hole feeding. In all specimens, the interactions present dark reaction rims variable in thickness, ranging from 0.05 mm to 0.2 mm.

DT04: specimen MAP-8353 (Figure 3f). Large circular perforation 9 mm long and 6 mm wide in the upper left area of the leaf, with smooth margins and lacking a reaction rim. The strings of veins are visible, as they are not consumed and they remain inside the hole.

DT07: specimen MAP-8355 (Figure 3g). Rectilinear elongate perforations 2 mm long and 0.3 mm to 0.7 mm wide, aligned and affecting several main veins with a distinct reaction rim present.

#### 3.1.2. Margin Feeding

DT12: specimens MAP-8347 (Figure 3d), MAP-8354 (Figure 3h), MAP-8356. Specimen MAP-8354 presents an excision in the whole left margin and also the upper right area of the leaf, with a crenulate pattern, <180 degrees of arc, and a distinct reaction rim.

DT13: specimen MAP-8348 (Figure 3i). Partial excision of the leaf apex concerning the left area and affecting the serrate margin where the teeth were erased. No reaction rim is present.

**Figure 4:**
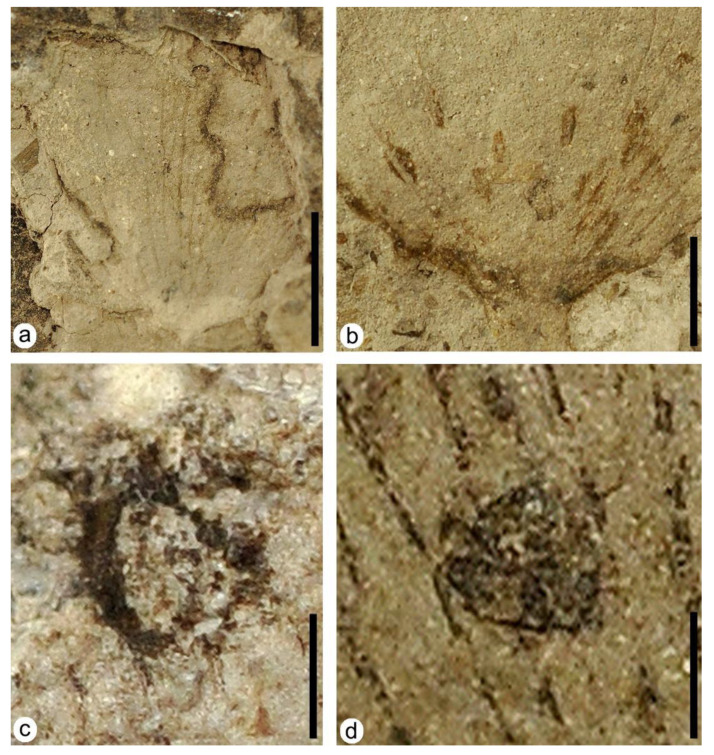
Plant–insect interactions on leaves of the aquatic angiosperm *Klitzschophyllites.* (**a**) MAP-8357, a nearly complete leaf with margin feeding DT15. (**b**) MAP-8354 detail of oviposition DT100 at base of the leaf from Figure 3h. (**c**) MAP-8347 detail of mining DT66 from Figure 3d showing tiny serpentiform coprolites in its internal area. (**d**) MAP-8349 detail of galling DT52 from Figure 3e. Scale bars: (**a**) = 1 cm; (**b**) = 5 mm; (**c**,**d**) = 0.5 mm.

DT15: specimens MAP-8357 (Figure 4a), MAP-8358, MAP-8355. In specimen MAP-8357, the leaf presents three margin feeding excisions, and two are placed at the left and right margin of the leaf, being 5.95 mm and 6.95 mm long, respectively. These two margin feeding excisions present a marked reaction rim of up to 0.65 mm in thickness. The shape of these interactions is irregular and penetrates deep into the leaf. The third margin feeding excision is located on the upper central margin of the leaf and forms an asymmetrical arc about 1.98 mm wide, with a subtle reaction rim. 

DT26: specimen MAP-8348 (Figure 3i). Removal of interveinal tissue in the lower left margin of the leaf with curved veinal stringers present.

DT81: specimen MAP-8349 (Figure 3e). The right edge of the leaf shows several crescentic herbivory excisions with a variable size, each with a marked, dark reaction rim (dark black arrowheads). At the lower right margin of the leaf, there are three continuous margin feeding excisions, of 0.55 mm, 0.68 mm and 1.28 mm in length respectively. The upper right margin exhibits a larger margin feeding excision 2.12 mm long with a softer reaction rim.

#### 3.1.3. Skeletonization

DT16: specimen MAP-8355 (Figure 3g). Interveinal tissue was removed in the central right area of the leaf with an irregular shape, following the direction of the main veins but with a reaction rim poorly developed.

#### 3.1.4. Oviposition

DT100: specimen MAP-8354 (Figure 3h and Figure 4b). Lenticular-shaped foliar scars (1 to 1.5 mm long and 0.2 to 0.5 mm wide) with acute endings in a cluster at the base of the leaf following adjacent linear files parallel to the main veins. Necrotic tissue is present in almost all of the scars.

#### 3.1.5. Mining

DT66: specimen MAP-8347 (Figure 3d and Figure 4c). A subcircular mine with an irregular shape located on the left margin of this leaf (Figure 3d, white arrowhead). Its size is about 0.77 mm by 0.78mm, and it presents a dark well-developed outer reaction rim with a variable thickness that ranges from 0.04 mm to 0.18 mm, containing 11 linear-shaped dispersed coprolites of variable length (Figure 4c) that range from 0.02 mm for the shortest coprolite to 0.15 mm for the longest coprolite. 

#### 3.1.6. Galling

DT52: specimen MAP-8349 (Figure 3e and Figure 4d). A subcircular structure, dark in color, with a distinctive relief that differentiates it from the rest of the leaf (Figure 3e, white arrowhead). The outer wall of the gall is 0.63 mm by 0.67 mm in diameter and is located along the central left part of the leaf, where it crosses some veins (Figure 4d).

### 3.2. Interactions on Leaves of Angiosperm Type 1

#### 3.2.1. Hole Feeding

**Figure 5:**
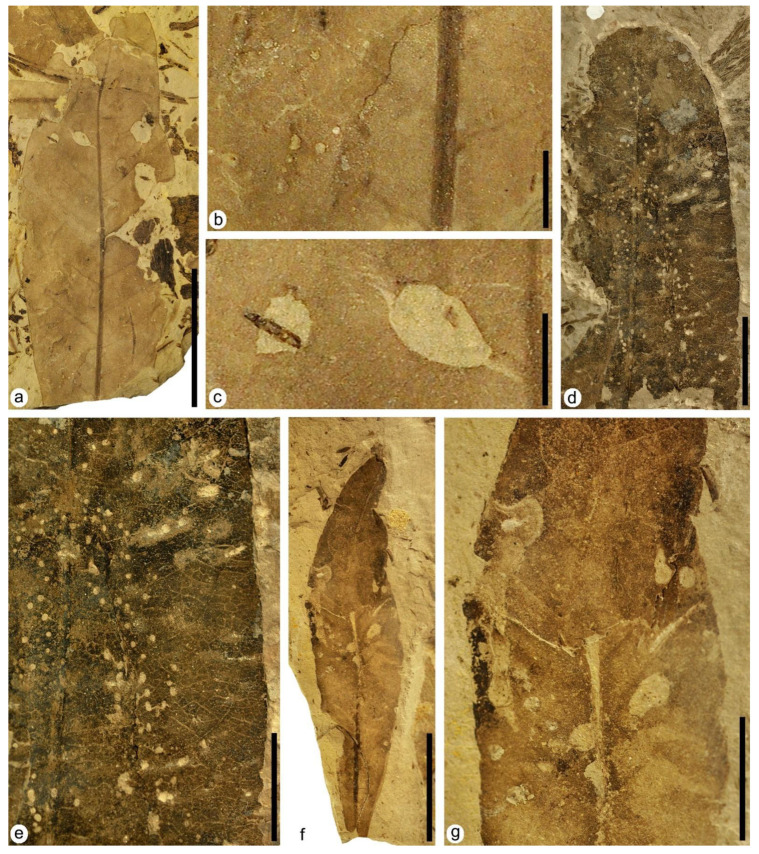
Plant–insect interactions on leaves of the terrestrial Angiosperm Type 1. (**a**) MAP-8359, apical part of a leaf with hole feeding DT01 and DT03 and margin feeding DT15. (**b**) Detail of small holes of hole feeding DT01 in photo (**a**). (**c**) Detail of big holes of hole feeding DT03 in photo (**a**). (**d**) MAP-8360, apical part of a leaf with hole feeding DT01. (**e**) Detail of small holes in photo (**d**). (**f**) MAP-8361, nearly complete leaf with hole feeding DT02 and DT03. (**g**) Detail of hole feeding DT02 and DT03 in photo (**f**). Scale bars: (**a**,**d**,**f**) = 2 cm; (**e**,**g**) = 1 cm; (**b**,**c**) = 5 mm.

DT01: specimens MAP-8359 (Figure 5a,b), MAP-8360 (Figure 5d,e). Small holes, which are circular to subcircular in shape, less than 1mm in size, ranging from 0.98 mm by 0.7 mm for the largest to 0.1 mm by 0.1 mm for the smallest holes (Figure 5a,b). The holes have a smooth-textured dark reaction rim about 0.1 mm wide and do not appear to follow any particular pattern in specimen MAP-8359 (Figure 5a,b) but follow a preferential orientation subparallel to the main vein in specimen MAP-8360 (Figure 5d,e).

DT02: specimens MAP-8361 (Figure 5f,g), MAP-8362. Holes are circular to subcircular in shape, from 1 mm to 2 mm in diameter, placed randomly along the leaf and show a light reaction rim.

**Figure 6:**
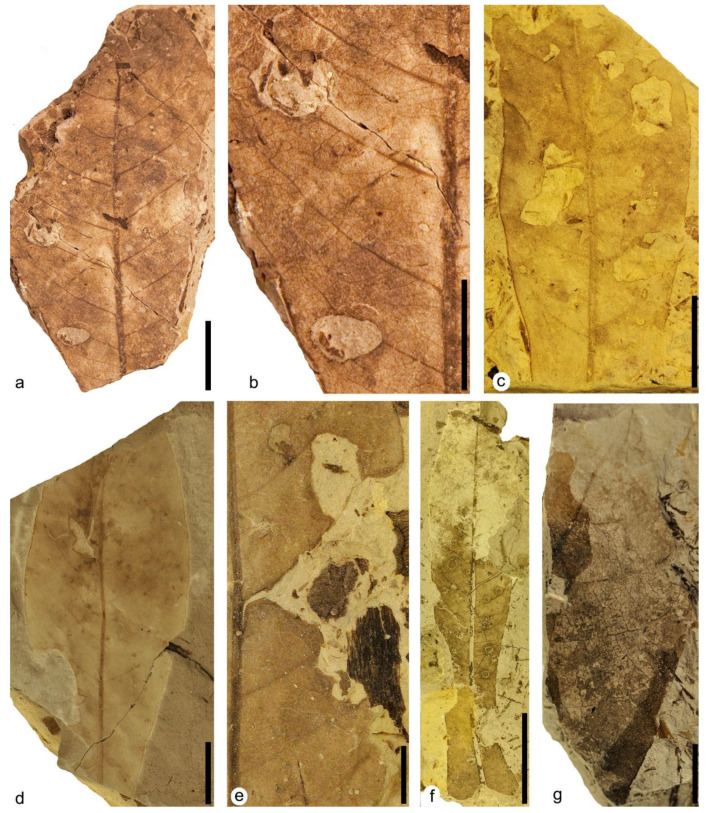
Plant–insect interactions on leaves of the terrestrial Angiosperm Type 1. (**a**) MAP-8362, fragment of a big leaf with hole feeding DT03 and DT04. (**b**) Detail of big holes of hole feeding DT03 and DT04 in photo (**a**). (**c**) MAP-8363, middle part of a leaf with hole feeding DT03, DT05 and margin feeding DT13. (**d**) MAP-8364, central part of leaf with hole feeding DT05, margin feeding DT14 and piercing and sucking DT46. (**e**) Detail of the right margin in the central area of the leaf in Figure 5a corresponding to MAP-8359 with margin feeding DT15. (**f**) MAP-8365, a nearly complete big leaf with skeletonization DT16 and galling DT120 bearing several galls distributed randomly on its surface. (**g**) MAP-8366, fragment of leaf with skeletonization DT17. Scale bars: (**a**,**c**,**d**,**f**) = 2 cm; (**b**,**e**,**g**) = 1 cm.

DT03: specimens MAP-8359 (Figure 5a,c), MAP-8361 (Figure 5f,g), MAP-8362 (Figure 6a,b). MAP-8363 (Figure 6c). Specimen MAP-8359 presents large holes of size >1 mm. The holes are polylobate to irregular-shaped, with sizes that range from 3.8 mm to 2.27 mm long. The distribution of these holes does not seem to follow any pattern, and they traverse secondary veins of the leaf.

DT04: specimen MAP-8362 (Figure 6a,b). Large holes subcircular in shape, up to 12 mm long and 9 mm wide. The lower left hole in the leaf presents small nearly symmetrical circular swallow excisions in its inner margin, and both holes display a weak reaction rim about 0.25 mm wide.

**Figure 7:**
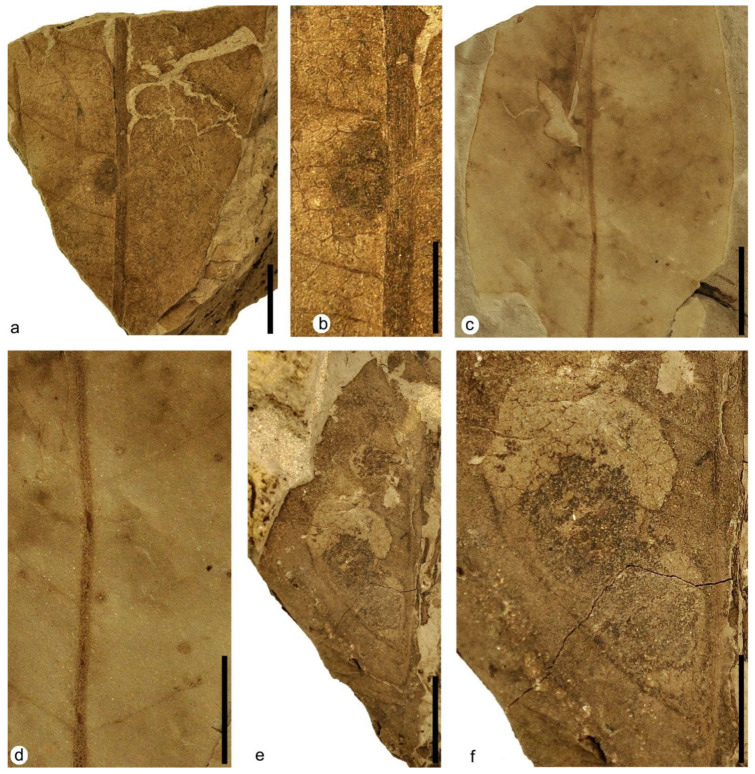
Plant–insect interactions on leaves of the terrestrial Angiosperm Type 1. (**a**) MAP-8367, fragment of leaf with surface feeding DT29. (**b**) Detail of surface feeding DT29 in photo (**a**). (**c**) Detail of the central area of the leaf in Figure 6df corresponding to MAP-8364 with hole feeding DT05, margin feeding DT14 and piercing and sucking DT46. (**d**) Detail of the central part of photo (**c**) with piercing and sucking DT46. (**e**) MAP-8368, a middle apical part of a leaf fragment with mining DT35 showing two complex mines. (**f**) Detail of the lower complex mine of photo (**e**) with mining DT35. Scale bars: (**a**,**c**,**d**,**f**) = 1 cm; (**b**) = 5 mm; (**e**) = 2 cm.

DT05: specimens MAP-8363 (Figure 6c), MAP-8364 (Figure 6d and Figure 7c). Polylobate perforations of size >5 mm in diameter. The holes present irregular shapes and a size that ranges from 8.5 mm long and 6.7 mm wide to 16 mm long and 10 mm wide. The holes present a light distinct reaction rim, about 0.1 mm to 0.2 mm wide. The distribution of these holes do not seem to follow any pattern and they are distributed randomly, crossing the secondary venation of the leaf at both sides of the main vein.

#### 3.2.2. Margin Feeding

DT13: specimen MAP-8363 (Figure 6c). Excision of leaf apex, including primary vein. The leaf has several feeding excisions along its margin, including the apex. At least six excisions are distinguishable. At the lower left margin, there is an excision approximately 16.9 mm in length, with a thin reaction rim that can be as wide as 0.2 mm. Two deep excisions up to 14.6 mm in depth and up to 13 mm in length can be seen in the upper right corner. The apex is absent, presenting an excision with a subtle reaction rim.

DT14: specimen MAP-8364 (Figure 6d and Figure 7c). Excision of the leaf to a primary vein in the mid-left zone that is 12 mm long and 7 mm wide erases several secondary veins but turns nearly perpendicular to the main vein at its endings. It presents a weak reaction rim.

DT15: specimen MAP-8359 (Figure 5a and Figure 6e). An excision that is deeply incised and expands toward the primary vein. At the upper right leaf edge, several excisions are observed, but the damage is so advanced in this area that it is difficult to determine the limits of each excision. These excisions affect 39.2 mm of the edge of the leaf and penetrate to a depth of approximately 17.3 mm and they have a slight dark reaction rim about 0.2 mm thick.

#### 3.2.3. Skeletonization

DT16: specimen MAP-8365 (Figure 6f). Interveinal tissue removed in the upper third of the leaf, with a poorly developed reaction rim. Most of the distal course of the secondary veins has been erased, but weakly tertiary and quaternary veins present in some areas as patches.

DT17: specimen MAP-8366 (Figure 6g). Interveinal tissue of the upper right area of the leaf has been removed, but almost all orders of veins are still well marked. The reaction rim is well developed.

#### 3.2.4. Surface Feeding

DT29: specimen MAP-8367 (Figure 7a,b). Removal or abrasion of surface tissues in an elliptical-shaped structure 4 mm long and 2.5 mm wide, dark brown in color, placed at the side of the main vein between two secondaries and with a weak reaction rim.

#### 3.2.5. Piercing and Sucking

DT46: specimen MAP-8364 (Figure 6d and Figure 7c,d). Circular punctures range from 1 mm to less than 2 mm in diameter, presenting a central depression and a distinct reaction rim. The punctures are distributed randomly on the surface of the leaf, and they do not affect the high-order veins.

#### 3.2.6. Mining

DT35: specimen MAP-8368 (Figure 7e,f). This leaf presents two complex blotch-shaped mines with a sinuous trajectory and containing coprolites of different sizes. The two mines are located on the left side of the leaf and appear to have a very similar structure, although they differ in size. The lower mine is larger, 26.61 mm by 12.58 mm, although it varies in width and is limited by a dark reaction rim up to 5 mm wide. At the beginning of the mine, the coprolites have a width of about 0.9 mm, but towards the end of the mine they are 3 mm wide.. In the upper part of the mine the leaf appears skeletonized, where the tissue between the secondary and tertiary veins was removed. Its reaction rim is dark and variable in thickness, from 0.1 mm to 0.2 mm. A darker lineation in its central area could correspond to an accumulation of coprolites (Figure 7f), but the sizes and detailed distribution of these structures cannot be adequately recognized.

**Figure 8:**
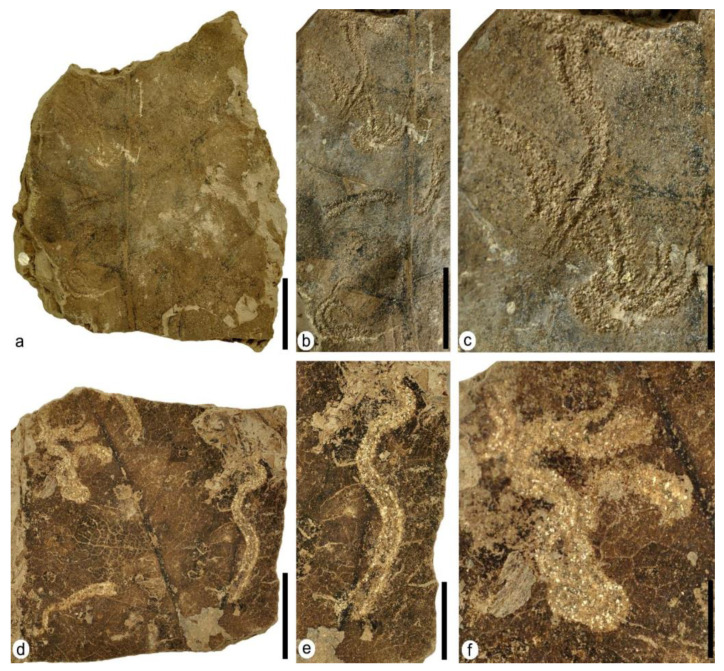
Plant–insect interactions—mining—on leaves of the terrestrial Angiosperm Type 1 with mining DT43 bearing serpentiform mines. (**a**) MAP-8369, fragment of a big leaf with several mines. (**b**) Detail of the left part of the leaf showing most of the mines from photo (**a**). (**c**) Detail of upper left part from photo (**b**) with two crossing mines bearing densely packed solid frass along the margins**.** (**d**) MAP-8370, another fragment of a big leaf with well-defined mines. (**e**) Detail of the right part from photo (**d**) showing a serpentine mine. (**f**) Detail of the upper left part from photo (**d**) showing a serpentine-convolute mine with both the margins and center covered by tiny coprolites. Scale bars: (**a**) = 2 cm; (**b**,**d**) = 1 cm; (**c**,**e**,**f**) = 5 mm.

DT43: specimens MAP-8369 (Figure 8a–c) and MAP-8370 (Figure 8d–f). Several serpentine-shaped mines spread over the entire surface of the leaves. The mines present different shapes, from slightly curved to highly sinuous to serpentine paths presenting thick, densely packed solid frass along the margins but a lower amount of frass in the center of the mine or lacking in some parts of this zone (Figure 8c,e). The mines present a relatively short length ranging between 8.5 mm and 18 mm, and their width is almost constant, at 1 mm in MAP-8369 and 1.5 mm in MAP-8370 on average in their middle zone and 0.3 mm and 0.7 mm at their ends, respectively. The mines present an apparent reaction rim, but the margins in almost all of them are covered by tiny coprolites 0.5 mm long and 0.15 mm wide on average.

#### 3.2.7. Galling

**Figure 9:**
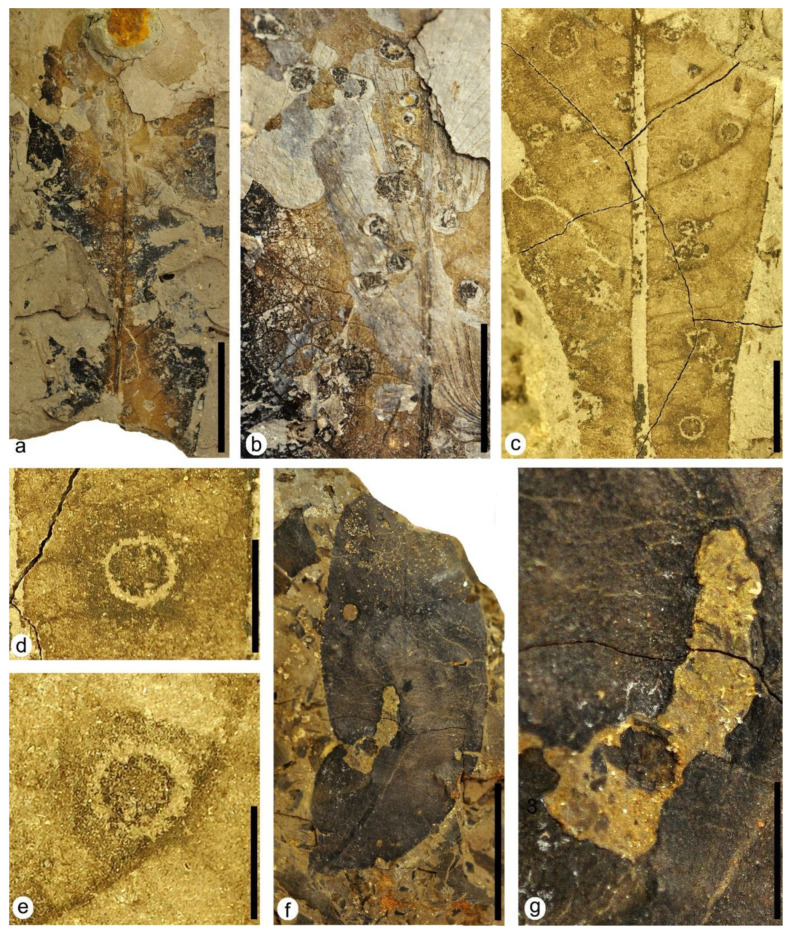
Plant–insect interactions on leaves of both the terrestrial Angiosperm Type 1 (photos **a**–**e**) and Angiosperm Type 2 (photos **f**,**g**). (**a**) MAP-8371, a highly degraded apical part of a leaf with galling DT62. (**b**) Detail of the upper right part of MAP-8371 from photo (**a**) with galling DT62 showing several galls bearing pockmarked to convoluted pustules disposed in chains in their interior. (**c**) Detail of the central area of MAP-8365 from Figure 6f with galling DT120 showing several galls on the lamina. (**d**,**e**) Details of two of the galls with galling DT120 from photo (**c**) showing the dark-colored patch around the light-colored inner circle. (**f**) MAP-8372, a nearly complete leaf of Angiosperm Type 2 with hole feeding DT01 and DT02 and margin feeding DT15. (**g**) Detail of the central left part of MAP-8372 from photo (**f**) showing margin feeding DT15. Scale bars: (**a**,**f**) = 2 cm; (**b**,**c**,**g**) = 1 cm; (**d**) = 5mm; (**e**) = 3 mm.

DT62: specimen MAP-8371 (Figure 9a,b). Flat, thickened, and circular-shaped structures from 0.7 mm to 2.5 mm in diameter are distributed randomly in the leaf. They present pockmarked to convoluted pustules disposed in chains in their interior, and some of them also present a distinct wide reaction rim.

DT120: specimen MAP-8365 (Figure 6f and Figure 9c–e). Subcircular to sub-polygonal structures between 1.7 mm and 3.3 mm in diameter, dark in color inside and preserving venation in this zone. The structures present an internal reaction ring 0.3 mm in width that is usually lighter in color but sometimes darker than the central zone of some galls (Figure 9c). The structures also show an encircled external patch subcircular to substellate in shape that is at least three times wider than the internal reaction ring, and it is darker in color than the surrounding tissue of the leaf (Figure 9d,e). The galls are arranged between the secondary veins of the leaf but, in some cases, they are attached to the margin of these veins (Figure 9c) and are organized in an isolated way or sometimes grouped in groups of three structures.

### 3.3. Interactions on Leaves of Angiosperm Type 2

#### 3.3.1. Hole Feeding

DT01: specimen MAP-8372 (Figure 9f). Several small holes circular in shape, ranging from 0.5 mm to nearly 1 mm in size, and showing a weak reaction rim. The holes do not appear to follow any particular distribution.

DT02: specimen MAP-8372 (Figure 9f). Two circular to subcircular perforations are 1.5 mm to 2 mm in diameter, with a distinct reaction rim near the margins of the leaf.

#### 3.3.2. Margin Feeding

DT15: specimen MAP-8372 (Figure 9f,g). A large elongated deep incision 10 mm long by 4 mm wide at the proximal zone and 2 mm wide at the distal zone. The incision penetrates up to the primary vein and then turns parallel to it. The margins of the incision are irregular in shape, showing a very distinct reaction rim that increases in width to the distal zone, reaching 0.6 mm thick.

## 4. Discussion

For the studied taxa containing plant–insect interactions, 23 different DTs were identified, belonging to eight FFGs assigned to hole feeding, margin feeding, skeletonization, surface feeding, piercing and sucking, mining, oviposition, and galling. The *Klitzschophyllites* leaves had 14 DTs, Angiosperm Type 1 leaves had 16 DTs, and Angiosperm Type 2 leaves had 3 DTs. This spectrum of DTs and FFGs represents a substantial diversity of damage for the specimens of these species, implying that a relatively wide variety of insects used *Klitzschophyllites* and Angiosperm Type 1 as host plants. The combined incidence of damage to these plants was 20.4%, which is not as high as that of other mid-Cretaceous floras [22,23].

The plant–insect interactions from the latest Albian in the Estercuel locality in Spain can be compared with those from the Rose Creek fossil site in the Dakota Formation in Nebraska, USA, due to the occurrence of angiosperm leaves in a coeval age [23]. Nevertheless, the types of interactions in the angiosperms from Nebraska very notably surpass the assemblage from Estercuel in both diversity, with 21 species/morphotypes, and abundance, with 2084 specimens studied. However, the assemblage from Estercuel surpasses some other coeval records in North America, including the assemblage of the Soap Flora from the Albian–Cenomanian deposits of the Cedar Mountain Formation in Utah in terms of the low rate, small to medium leaf area consumed, and also in the low diversity of damage type that is similar to the records from the Late Cretaceous [24].

The serpentine-shaped mines in MAP-8369 and MAP-8370 (Figure 8) are very similar to those described in [25] (Figures 4k and 5e,f) in leaves of angiosperms from the Paleocene of Montana, USA, including the parallel thick frass trails that are densely packed along the margins but diminished or lacking in the center of the mine.

The records of the plant–insect interactions in the leaves of *Klitzschophyllites* from the Boundary Unit over the Utrillas Formation in Estercuel have also been found in the Aptian–Albian deposits of the Crato Formation in northeastern Brazil studied by Filho et al. [13], which was the only work at the time that had studied interactions on leaves of this taxon. Notably, the interactions on the leaves of this genus from Spain were much more diverse and abundant, with 14 damage types corresponding to eight feeding groups, than those in the Brazilian records, with two types from two feeding groups, indicating substantial paleoenvironmental and paleoclimatic differences between these two zones in mid-Cretaceous times. Nevertheless, the difference concerning the diversity of damage seen in the Spanish fossils as opposed to the Brazilian ones may be due to the larger sample of the former, and also because damaged leaves from Crato beds were in the past discarded by collectors who considered them poorly preserved. However, although both the Crato Formation and the Boundary Marls Unit over the Utrillas Formation are related to coastal paleoenvironments, the Brazilian deposits correspond to semiarid to arid conditions [26]. In fact, the high diversity of plant–insect interactions in Estercuel indicate more humid paleoenvironmental conditions in this zone of the Tethys realm during the latest Albian.

Oviposition was found exclusively in a single leaf of *Klitzschophyllites* from Estercuel (Figure 3h and Figure 4b) and followed the same distribution pattern of oviposition scars in some leaves of the aquatic angiosperm *Quereuxia angulata* (Lesq.) Krysht. from the Upper Cretaceous of the Amur Region in Russia [27]. These leaves present a morphology and venation pattern at the base of the lamina very similar to those in leaves of *Klitzschophyllites* from Estercuel, as it also presents similarities in the morphology and distribution of the oviposition marks at the base of the leaves ([27], Figure 2b,c). This kind of interaction—oviposition—in leaves of *Quereuxia angulata* was classified as *Paleoovoidus arcuatus* Vasilenko, and was assigned to Odonata, probably produced by dragonflies of the suborder Anisoptera due to the distribution pattern of the eggs on the leaves [28].

The sedimentological interpretation of the studied fossil site of Estercuel indicated a coastal lacustrine environment with fluvial input and tidal influence [16,19], where the angiosperms inhabited the shores of small masses of stagnant freshwater bodies such as ponds. In this context, a variety of adult insects would use these plants to feed and leave their eggs and immatures, such as leaf miners, to develop. Those insects that inhabited these partially submerged plants would have the ability to move through air or water; putative culprits could be members of the orders Odonata (dragonflies and damselflies), Ephemeroptera (mayflies), Plecoptera (stoneflies), Trichoptera (caddisflies), Hemiptera (e.g., aphids or bugs), Lepidoptera, some Coleoptera, or Diptera (e.g., flies) that had an aquatic larval development, with adults tending to remain near water. Concerning these animals that produce interactions in leaves, several types of arthropods were also found in the middle to upper Albian amber deposits from the Iberian Peninsula, including Arachnida and insects such as Collembola, Blattodea, Orthoptera, Psocoptera, Archaeognatha, Hemiptera, Thysanoptera, Lepidoptera, Diptera, Coleoptera, Neuroptera and Hymenoptera [28,29,30,31].

The different damage types of external feeding found in the specimens studied (such as margin feeding or hole feeding) could be produced by a broader variety of insects including Orthoptera, Coleoptera, and maybe larval Lepidoptera. Previously, also in the latest Albian of Spain, Estévez-Gallardo et al. [12] found evidence of hole feeding and margin feeding in *Ploufolia cerciforme* and *Aquatifolia fluitans* (Nymphaeales), which are both aquatic plants presenting morphological structures and adaptations similar to those found in the leaves of *Klitzschophyllites*. However, the high generalist specificity of these types of interactions does not allow an association with a particular family of insects. 

Alternatively, mines, galls and even some types of “piercing and sucking” were more specialized interactions than external feeding damage, such as margin feeding, hole feeding, surface feeding or skeletonization. In the case of the gall found in *Klitzschophyllites* (interpreted as DT52), it was assigned the maximum value of host specificity in [32] because the presence of galling in an assemblage with an aquatic plant is necessarily in contrast with the current preferences of gall-inducing insects for xeric environments [33,34].

In extant floras, the principal group of gall-inducing insects belongs to Diptera, specifically the Cecidomyiidae (gall midges), followed by Hymenoptera, mainly Cynipidae (gall wasps), Hemiptera (mostly aphids and scale insects), and Thysanoptera (thrips) [34]. Nonetheless, the galls found in *Klitzschophyllites* could be due to non-insect organisms, since galls of these dimensions and morphologies also could be caused by groups such as Chromista protists, mites, or fungi [35]. For the Aptian-Albian Crato Fm., *Klitzschophyllites* also presents evidence of galling [13], so it appears that the preference of gall-inducing insects for these basal aquatic angiosperms could be a more general phenomenon than expected. In extant ecosystems, dicots have a higher risk of attack by gall-inducing insects than monocots [36]. The evidence of galling in the aquatic angiosperm *Klitzschophyllites* and in leaves of the terrestrial Angiosperm Type 1 suggests that this closer relationship between gall-inducing insects and dicots had already existed by the latest Albian.

Evidence for mining in *Klitzschophyllites* is similar to the example for DT66 in [32]. However, this type of damage may be considered alternatively as that of a pathogen, causing citrus canker affecting multiple genera in the family Rutaceae, and is caused by the modern gammaproteobacterium *Xanthomonas axonopodis* var. *citri* (Xanthomonadales: Xanthomonadaceae) (C.C. Labandeira pers. communication, 2022). Nevertheless, the structure and shape of the mine show significant similarities with the mines produced by living Lepidoptera of the Gracillariidae family (leaf-mining moths) such as Phyllonorycter (see Figure 17.7b in López-Vaamonde [37]) or mines produced by some species of the gracillariid *Philodoria* (see Figure 10 in Kobayashi [38]). The reported plant–insect associations on these semi-submerged leaves represent “safe” habitats that lepidopteran larvae and other insects could inhabit, use to feed, and defend from predators. Concerning this fossil evidence from Estercuel, it is notable that direct remains of lepidopterans were found inside amber in deposits from the Albian of the Iberian Peninsula [28], and the presence of the superfamily Gracillaroidea has recently been reported in the Albian of Myanmar [39].

One example of the mining evidence found in Angiosperm Type 1 specimen MAP-8368 (Figure 7e,f) presents a more complex structure than the mine in *Klitzschophyllites,* and it is more common in extant floras. This type of mine could have been produced by several species of different insect orders, including Lepidoptera, Hemiptera, or possibly Diptera, all represented in the Albian amber of the Iberian Peninsula [38].

The plant–insect interactions studied here show that early angiosperms were heavily herbivorized by insects or other arthropods during the latest Albian. These high herbivory rates in angiosperms are consistent with other studies of plant–insect interactions from the mid-Cretaceous of France [40], which have also shown a clear preference of insects for early angiosperms over other groups of plants such as gymnosperms or ferns. Therefore, this preference could have been more generalized in mid-Cretaceous ecosystems than previously assumed. Future studies on plant–insect interactions in Aptian-Albian floras from the Iberian Peninsula, including pteridophytes, gymnosperms, and angiosperms, could allow us to understand better the dynamics of plant–insect interactions during the Cretaceous Terrestrial Revolution and the changes in herbivory patterns for the main groups of plants.

## 5. Materials and Methods

For the present work, 142 angiosperm leaves from the latest Albian in the “La Dehesa” locality near Estercuel (Spain) were examined. Only specimens with evident plant–insect interactions—27 specimens—were selected for the study, 13 of which corresponded to leaves of genus *Klitzschophyllites* (specimens MAP-8346 to MAP-8358) 13 specimens were Angiosperm Type 1 (specimens MAP-8359 to MAP-8371), and one specimen was identified as Angiosperm Type 2 (specimen MAP-8372). All selected samples were stored at the Museo Aragonés de Paleontología (MAP) at Fundación Conjunto Paleontológico de Teruel-Dinópolis (Teruel City, Spain).

The fossil leaves were prepared using a micro-pneumatic hammer and sharp needles under a stereomicroscope to remove the matrix material covering the fossils. Photographs were taken using a Nikon D-90 camera with an AF-S Micro Nikkor 60 mm macro lens.

We used the classification system proposed by [32] to identify plant-arthropod interactions, in which the damages observed in the leaves are classified into different morphotypes, termed damage types (DTs), and given an identifying number where the DTs belong to a particular functional feeding group (FFG). Additionally, each type of DT was identified with a certain degree of specialization based on its distribution on different taxa of host plants.

## 6. Conclusions

The presence of 23 damage types belonging to eight different functional feeding groups in early angiosperms from the latest Albian in Spain suggests a close relationship between insects and this group of plants. This evidence points out that angiosperms were an essential source of food and lodging for insects in the Iberian ecosystems during the late Early Cretaceous in the southwestern zone of the Tethys realm. In addition, basal aquatic eudicots, such as *Klitzschophyllites*, seem to represent a food source and a host place for different groups of arthropods, including certain groups of lepidopteran insects, probably members of the Gracillariidae (leaf-mining moths). 

## Data Availability

Not applicable.
